# Phenotypic Screens Identify Parasite Genetic Factors Associated with Malarial Fever Response in *Plasmodium falciparum piggyBac* Mutants

**DOI:** 10.1128/mSphere.00273-16

**Published:** 2016-10-26

**Authors:** Phaedra Thomas, Jennifer Sedillo, Jenna Oberstaller, Suzanne Li, Min Zhang, Naresh Singh, Chengqi C. Q. Wang, Kenneth Udenze, Rays H. Y. Jiang, John H. Adams

**Affiliations:** Center for Global Health and Infectious Diseases Research, Department of Global Health, University of South Florida, Tampa, Florida, USA; University of Texas Southwestern

**Keywords:** forward genetics, heat shock, phenotype screen, *piggyBac*, transposon-mediated mutagenesis, virulence factors

## Abstract

Malaria remains one of the most devastating parasitic diseases worldwide, with 90% of the malaria deaths in Africa in 2013 attributable to *Plasmodium falciparum*. The clinical symptoms of malaria include cycles of fever, corresponding to parasite rupture from red blood cells every 48 h. Parasite pathways involved in the parasite’s ability to survive the host fever response, and indeed, the functions of ~40% of *P. falciparum* genes as a whole, are still largely unknown. Here, we evaluated the potential of scalable forward-genetic screening methods to identify genes involved in the host fever response. We performed a phenotypic screen for genes linked to the parasite response to febrile temperatures by utilizing a selection of single-disruption *P. falciparum* mutants generated via random *piggyBac* transposon mutagenesis in a previous study. We identified several mutants presenting significant phenotypes in febrile response screens compared to the wild type, indicating possible roles for the disrupted genes in this process. We present these initial studies as proof that forward genetics can be used to gain insight into critical factors associated with parasite biology.

**IMPORTANCE** Though the *P. falciparum* genome sequence has been available for many years, ~40% of its genes do not have informative annotations, as they show no detectable homology to those of studied organisms. More still have not been evaluated via genetic methods. Scalable forward-genetic approaches that allow interrogation of gene function without any pre-existing knowledge are needed to hasten understanding of parasite biology, which will expedite the identification of drug targets and the development of future interventions in the face of spreading resistance to existing frontline drugs. In this work, we describe a new approach to pursue forward-genetic phenotypic screens for *P. falciparum* to identify factors associated with virulence. Future large-scale phenotypic screens developed to probe other such interesting phenomena, when considered in parallel, will prove a powerful tool for functional annotation of the *P. falciparum* genome, where so much remains undiscovered.

## Abstract (precis)

Though the *P. falciparum* genome sequence has been available for many years, ~40% of its genes do not have informative annotations, as they show no detectable homology to those of studied organisms. More still have not been evaluated via genetic methods. Scalable forward-genetic approaches that allow interrogation of gene function without any pre-existing knowledge are needed to hasten understanding of parasite biology, which will expedite the identification of drug targets and the development of future interventions in the face of spreading resistance to existing frontline drugs. In this work, we describe a new approach to pursue forward-genetic phenotypic screens for *P. falciparum* to identify factors associated with virulence. Future large-scale phenotypic screens developed to probe other such interesting phenomena, when considered in parallel, will prove a powerful tool for functional annotation of the *P. falciparum* genome, where so much remains undiscovered.

## INTRODUCTION

Malaria is one of the most devastating parasitic diseases worldwide; it was responsible for an estimated 198 million clinical cases and 584,000 deaths in 2013 alone (1). Ninety percent of the deaths due to malaria in Africa are attributed to *Plasmodium falciparum*. *P. falciparum* has a complex life cycle in the human host, spanning stages in the liver and blood, the latter of which is responsible for the clinical manifestation of malaria. Clinical symptoms of the disease include a pattern of fever, chills, sweating, and rigor or shivering (2). Fever onset is a reaction to the late stages of the parasite, schizonts, rupturing to be released from nutrient-depleted and malformed red blood cells (RBCs). Elevated body temperature effectively kills any remaining parasites that are not in the early stages of development (the ring stage) and synchronizes invasion (3, 4). Though there is some decline in parasitemia as a result of host fever, the parasite still manages to escape total destruction and complete its life cycle through mechanisms that are not well understood. Heat shock proteins (HSPs) may play a significant role in proteostasis during febrile episodes (5, 6), as a temperature of 41°C leads to the unfolding of proteins and *Plasmodium* has an expanded repertoire of HSP partner DnaJ domain-containing proteins, many of which are upregulated in response to febrile temperature stress (4). Previous studies have also indicated that *P. falciparum*-infected RBCs (iRBCs) undergo temperature-related membrane stiffening that may aid in parasite survival, implicating factors important for remodeling of the host cell, as well as structural proteins, in the heat shock response (4, 7).

Parasite pathways involved in the parasite’s ability to survive the host fever response are still largely unknown. Functional information about the genome as a whole is still sparse, with ~40% of the 5,777 *P. falciparum* genes having uninformative annotations (see http://www.plasmodb.org). Traditional targeted, reverse-genetic approaches have limited utility in a system where the functions of so many genes remain unknown. Forward-genetic approaches that allow us to interrogate gene function without any pre-existing knowledge are needed to hasten understanding of parasite biology, which will expedite the identification of drug targets and the development of future interventions.

Here, we describe an *in vitro* assay that we developed that is suitable for forward-genetic screens to identify genes involved in the important virulence process of the fever response by utilizing a selection of *P. falciparum* mutants generated via random *piggyBac* transposon mutagenesis in a previous study (8). *piggyBac* mutants are genetically identical, save a single genetic lesion where the transposon inserts itself randomly into the genome at TTAA tetranucleotide sites (9, 10). Mutants presenting significant phenotypes in febrile response screens compared to wild-type parasites implicate the disrupted gene’s involvement in this process. We present these initial studies as proof of the utility of forward-genetic analysis of *piggyBac* insertional mutants to gain insight into parasite biology.

## RESULTS

### *piggyBac* mutants subjected to phenotypic screens.

We used a random selection of 25 single-insertion *P. falciparum piggyBac* mutant clones from a previously described mutant library (8, 10), as well as the wild-type parent NF54 to associate altered phenotypic effects of febrile temperature with specific genotypes (Table 1). *piggyBac* insertions were first identified by thermal asymmetric interlaced (TAIL) PCR for transposon insertion identification (11) and verified by quantitative insertion site sequencing (QISeq) (10). Several mutants were additionally verified by whole-genome sequencing (WGS; Table 1) to ensure that no major genomic changes occurred aside from the *piggyBac* insertion. Selected mutants reflect disruptions in genes spanning a range of functional categories, as well as many genes without existing functional information.

**TABLE 1:** *piggyBac* mutants included in phenotypic screens^a^

*piggyBac* ID	Closest gene ID	Gene description	Strand	Type of insertion	Distance to gene (bp)	Clone	PCR	QIseq	WGS
PB-43	PF3D7_0206100	Cysteine desulfuration protein SufE	+	3′ UTR	−293	X	X	X	X
PB-9	PF3D7_0404600	Conserved *Plasmodium* membrane protein, unknown function	+	Exon	0	X	X	X	
PB-11	PF3D7_0416500	Repressor of RNA polymerase III transcription MAF1, putative	−	Exon	0	X	X	X	
PB-14	PF3D7_0511500	RNA pseudouridylate synthase, putative	+	Exon	0	X	X	X	X
PB-17	PF3D7_0521900	Conserved *Plasmodium* protein, unknown function	+	Exon	0	X	X	X	X
PB-3	PF3D7_0615900	Conserved *Plasmodium* protein, unknown function	−	Exon	0	X	X	X	X
PB-125	PF3D7_0622900	Transcription factor with AP2 domain(s), putative (ApiAP2)	−	Intergenic	−2,407	X	X	X	
PB-20	PF3D7_0808700	Erythrocyte membrane protein 1, PfEMP1 (VAR)	+	Intron	0	X	X	X	
PB-1	PF3D7_0811300	CCR4-associated factor 1 (CAF1)	+	Exon	0	X	X	X	X
PB-54	PF3D7_0902200	Serine/threonine protein kinase, FIKK family (FIKK9.3)	−	Exon	0	X	X	X	
PB-22	PF3D7_0931000	Elongation factor Tu, putative	−	Exon	0	X	X	X	
PB-12	PF3D7_1018300	Conserved *Plasmodium* protein, unknown function	−	Intergenic	−834	X	X	X	
PB-25	PF3D7_1035800	Probable protein, unknown function (M712)	+	Exon	0	X	X	X	X
PB-4	PF3D7_1122900	Dynein heavy chain, putative	+	Exon	0	X	X	X	
PB-19	PF3D7_1133700	Conserved *Plasmodium* protein, unknown function	+	Exon	0	X	X	X	X
PB-21	PF3D7_1136000	Conserved *Plasmodium* protein, unknown function	+	Exon	0	X	X	X	X
PB-28	PF3D7_1138900	ncRNA/unspecified product	+	Exon	0	X	X	X	
PB-5	PF3D7_1141900	Inner membrane complex protein 1b, putative (IMC1b)	−	Exon	0	X	X	X	
PB-33	PF3D7_1207800	Conserved *Plasmodium* protein, unknown function	−	Exon	0	X	X	X	X
PB-18	PF3D7_1219300	Erythrocyte membrane protein 1, PfEMP1 (VAR)	−	Exon	0	X	X	X	
PB-24	PF3D7_1231800	Asparagine-rich protein, putative	+	Exon	0	X	X	X	
PB-2	PF3D7_1305500	MAPK phosphatase 1, putative (MKP1)	+	Exon	0	X	X	X	X
PB-58	PF3D7_1343700	Kelch protein K13	−	Intergenic	−1,034	X	X	X	
PB-120	PF3D7_1459500	Conserved *Plasmodium* protein, unknown function	+	5′ UTR	+244	X	X	X	
PB-6	PF3D7_1475700	Tubulin epsilon chain, putative	+	Intergenic	+908	X	X	X	

a Insertion positions were determined by QISeq. Positive distances indicate insertions upstream of the translation start site. Negative distances indicate insertions downstream of the translation stop site. Validation of mutants included limiting-dilution cloning, TAIL PCR and QIseq to verify the transposon insertion site, and WGS of select mutants to confirm that there were no other major genomic changes.

### Development of a scalable heat shock screen.

**FIG 1:**
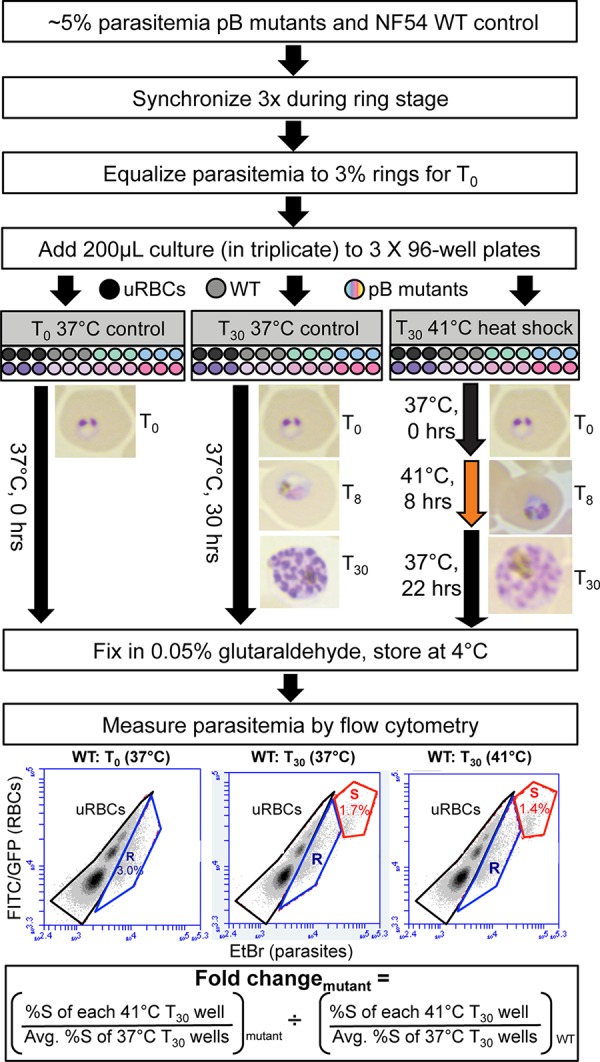
Heat shock assay procedure. Shown is the setup for the heat shock screen as conducted with 96-well plates. *T*_0_ plates represent the ring stage or the beginning of the experiment, *T*_30_ plates at 37°C represent the controls, and *T*_30_ plates at 41°C are the hyperthermic-temperature-treated samples. Three technical and three biological replicates were performed for each well. Fixed wells are stained with fluorescein isothiocyanate (FITC) to detect RBCs and ethidium bromide (EtBr) to detect parasites via flow cytometry. Fold change was determined by using the formula shown at the bottom for each *T*_30_ 41°C well, averaged across three biological replicates. R, rings; S, schizonts; uRBCs, uninfected RBCs; %S, percent schizont parasitemia; WT, wild type. See Materials and Methods for full details.

*P. falciparum piggyBac* mutant parasites were subjected to heat shock stress *in vitro* to investigate factors responsible for maintaining growth under fever-like conditions (our methodology is summarized in Fig. 1). The basis of this analysis is a previously published study involving *P. falciparum* wild-type cultures exposed to hyperthermic temperatures (4). Elevated temperature inhibits the growth of the parasite, especially at the trophozoite and schizont stages, whereas ring stage parasites survive at 41°C for up to 24 h. Since there is not a severe drop in ring stage parasite viability until after 8 h, we used one round of heat shock at 41°C for 8 h to simulate a fever episode in our heat shock phenotype screen, with the cultures returning to 37°C or core body temperature for the remainder of the life cycle. NF54 wild-type and mutant cultures were left to grow after heat shock until 30 h, monitored via microscopy. Though parasites continued to grow after heat shock, they displayed signs of cellular crisis with less segmentation of the nuclei at the schizont stage, as previously noted (4), potentially leading to pyknotic forms (Fig. 1).

**FIG 2:**
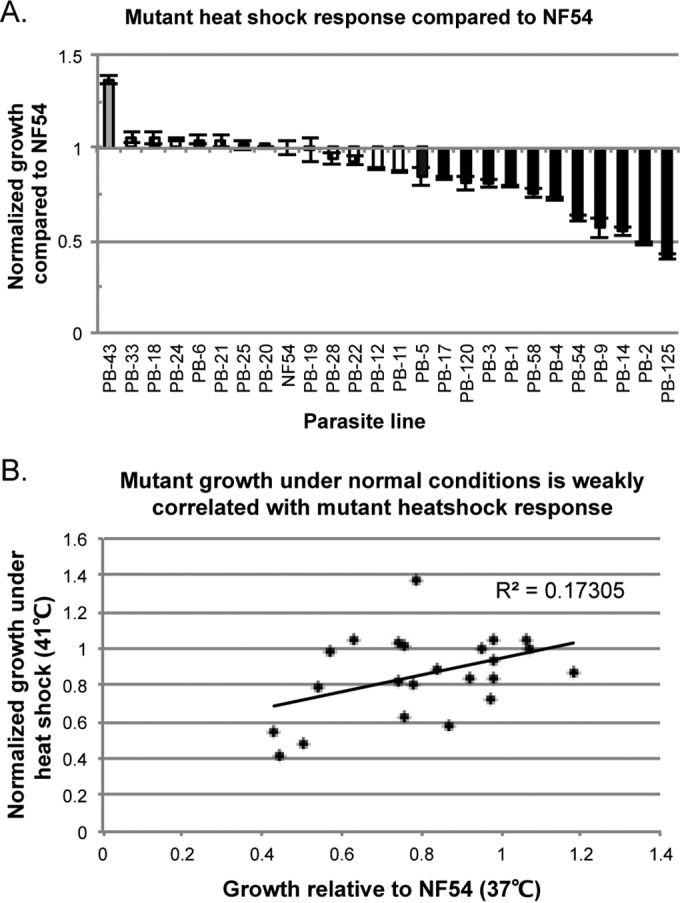
Heat shock response screen of *piggyBac* mutants. (A) *P. falciparum* clone NF54 and mutants (each PB ID number indicates the locus of transposon insertion; see Table 1) are shown on a graph according to normalized growth in response to febrile temperature stress compared to that of the NF54 parent line. Each mutant’s growth under heat shock was normalized to its growth at 37°C to account for inherent differences in growth. Gray bars indicate mutants with increased growth in response to heat shock compared to that of NF54, white bars indicate mutants that are not significantly different from NF54, and black bars indicate mutants with significantly decreased growth. Three technical replicates and three independent biological replicates were completed for each mutant line. Error bars indicate the standard error of the mean of biological replicates. Statistical significance was determined by one-way ANOVA at *P* < 0.05 for individual experiments. (B) Mutant fold change in growth at 41°C plotted against the mutant growth rate at 37°C (as determined by Balu et al. [11]). Mutant growth defects are weakly correlated with heat shock response phenotypes.

Three technical replicates and three biological replicates were prepared for each mutant, the wild-type NF54 control, and each temperature condition (37°C and 41°C). At the conclusion of the assay, parasites were fixed with glutaraldehyde and parasitemia was determined via flow cytometry by previously established methods (11). As several tested *piggyBac* mutants display attenuated growth compared to the wild type (11), the fold change in the growth of each parasite after heat shock compared to each parasite’s growth at 37°C was determined to control for growth differences not due to heat shock. Mutants with statistically significant standardized fold changes in growth compared to wild-type NF54 were determined via one-way analysis of variance (ANOVA) (Fig. 2A), and fold changes in growth were highly reproducible.

### Heat shock induces growth changes in *piggyBac* mutants.

Twelve of the 25 mutants had a statistically significant decrease in growth after heat shock. In agreement with previous predictions of processes involved in the heat shock response based on altered transcriptional profiles in heat-shocked parasites versus control parasites (4), mutants with attenuated growth after heat shock had disruptions in genes with annotations or gene ontology (GO) terms associated with the membrane or structural maintenance of the cell (PB-9, PB-4, PB-5, PB-120), transcription or RNA metabolism (PB-14, PB-125, PB-1), cell signaling (PB-2), or remodeling of the host cell (PB-54) (Table 1; Fig. 2A). Interestingly, mutant PB-58, which has an intergenic insertion that results in upregulation of the K13 propeller gene (PF3D7_1343700) (12) and has recently been implicated in rising resistance to the frontline drug artemisinin (13), had significantly increased sensitivity to heat shock. The remaining decreased-growth mutant disrupted genes (PB-17, PB-3) have uninformative annotations, and there is little available functional information about them, though sparse GO terms suggest involvement in motor activity and catalytic activity, respectively. Though our results support many of the core processes previously implicated in the heat shock response, there is very little overlap between genes we identified and existing data.

One mutant (PB-43, with a disruption in the 3′ untranslated region [UTR] of the apicoplast-targeted cysteine desulfuration protein SufE-encoding gene) demonstrated an increase in growth after heat shock (Table 1; Fig. 2A). The remaining parasites had no significant change in growth and are largely conserved proteins of unknown function, with some involved in transcription or translation. Though previous studies have implicated *var* genes in the febrile temperature response (4, 7), the two *var* gene mutants in our study had no phenotype, perhaps because these genes were not expressed in our parasites.

As several of the 25 mutants displayed attenuated growth phenotypes compared to that of NF54 under normal conditions (11), we evaluated the correlation between the pre-existing growth phenotype and the growth phenotype after heat shock. We found these phenotypes to be only weakly correlated (*R*^2^ = 0.17305; Fig. 2B), suggesting that the pre-existing growth phenotype is not sufficient to explain the heat shock response phenotype.

### Complementation reverses the heat shock response phenotype.

**FIG 3:**
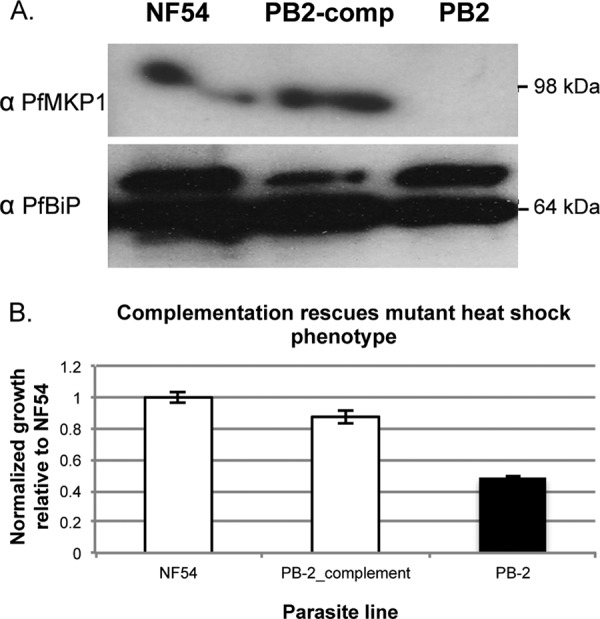
Complementation reverses the mutant heat shock phenotype. (A) Western blot assay of parasite lysate showing expression of PfMKP1 in the wild-type and complemented mutant strains but not in the mutant PB-2 strain. PfBip is shown as a loading control. (B) *P. falciparum* clone NF54, the complemented PB-2 mutant, and the PB-2 mutant are shown on a graph according to normalized growth in response to febrile temperature stress as in Fig. 2. The complemented PB-2 mutant displayed growth after heat shock that was restored to wild-type NF54 levels. Error bars indicate the standard error of the mean of three biological replicates. Statistical significance (black bar) was determined by one-way ANOVA at *P* < 0.05 for individual experiments.

One of the mutants (PB-2) that showed a decrease in growth in response to heat shock contains a *piggyBac* transposon insertion in the PF3D7_1305500 (PfMKP1) gene (Table 1; Fig. 2). To establish whether the heat shock phenotype was directly due to insertion in the PfMKP1 coding sequence, the PB-2 mutant was complemented with an episomal copy of the PfMKP1 gene with its native 5′ UTR and a 3′ three-hemagglutinin tag, followed by an HRP3 3′ UTR. Reverse transcription-PCR previously confirmed that the complemented line reintroduced the expression of PfMKP1 RNA (14), and PfMKP1 protein expression was confirmed in wild-type NF54 and the complemented mutant by Western blotting with a custom monoclonal antibody (anti-PfMKP1), while the PB-2 mutant showed no expression (Fig. 3A). To confirm that the decreased growth after heat shock was due to the loss of PfMKP1 expression, the heat shock assay was performed with the PfMKP1-complemented line. Complementation of PfMKP1 fully restored the heat shock phenotype to wild-type levels (Fig. 3B).

## DISCUSSION

Virulence factors allowing the parasite to survive the host fever response are unique parasite attributes paramount for survival in the host. These attributes make understanding pathways involved in fever responses of great interest for drug discovery, particularly in the face of rising resistance and treatment failure (15). The dearth of functional information about much of the genome complicates efforts to design targeted assays to study facets of parasite biology that might inform drug development. Here, we demonstrated the utility of *piggyBac* transposon-mediated random insertional mutagenesis for functional profiling of the *P. falciparum* genome by using phenotypic screens, with no pre-existing knowledge of gene function required. We developed scalable methods for identifying parasite factors involved in the febrile temperature response. We applied these methods to a small library of *piggyBac P. falciparum* mutants with random transposon insertions in or near genes, many of which have no homologs in other eukaryotes (11). This work represents the first application of *piggyBac* forward genetic screens to the study of virulence factors of malaria parasites.

**FIG 4:**
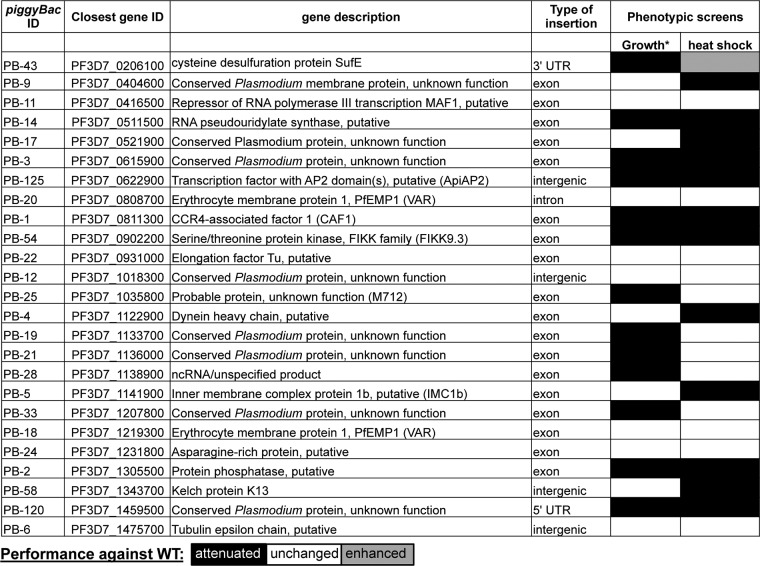
*piggyBac* mutant performance in a phenotypic screen. The performance of each mutant was compared with that of wild-type (WT) NF54 in growth and heat shock screens. Growth was determined as described by Balu et al. (11).

Though the *piggyBac* system is a powerful tool to study gene function, some factors should be considered in interpreting results. *piggyBac* methodology introduces a single random disruption per genome. The *piggyBac* transposon preferentially inserts itself at TTAA tetranucleotide sites, which can occur in any part of the gene body, or in intergenic regions. While insertions that occur in the coding sequence are likely to ablate that gene’s function, insertions that occur in predicted intergenic regions, introns, 5′ UTRs, or 3′ UTRs may have disparate or no consequences for the affected gene. Thus, while the transposon integration site has been precisely identified for each mutant, further studies are required to know the effects of the insertion on gene expression and protein production. Nonexonic insertion mutants not having a phenotype in our screens do not necessarily indicate that the affected gene has no role in the fever response; the insertion may just not have an effect on the gene. The vast majority of our screened mutants were exonic mutants (17 of 25; Table 1; Fig. 4), though mutants of all insertion types (except the lone intronic mutant) had interesting phenotypes.

Thirteen of our tested mutants have growth attenuation compared to the wild type under normal conditions (11), and seven of these mutants have decreased growth under heat shock conditions as well (Fig. 4). We found a weak correlation between growth attenuation and the heat shock phenotype (perhaps because growth is the metric for determining the heat shock response), even after heat-shocked mutants had been normalized to their own growth at 37°C to correct for the existing growth phenotype. One mutant with an insertion in a gene with no functional information displayed no growth phenotype but was attenuated in heat shock (PB-17). Four other mutants displayed phenotypes only in the heat shock screen (PB-4, PB-5, PB-9, PB-58). Taken together, these results support the specificity of our phenotype detection.

Previous studies have implicated membrane and structural proteins and proteins involved in transcription, RNA metabolism, cell signaling, and remodeling of the host cell in the heat shock response (4). Mutants with decreased growth compared to the wild type in our heat shock response screens largely belong to these categories (9 of 12 mutants). The relatively small sample size screened does not allow us to evaluate statistical enrichment, though the agreement between our data sets and the high reproducibility within our data suggest the validity of our approach. Though our results support many of the core processes previously implicated in the heat shock response, there is little overlap between individual genes. We additionally suggest a role in the heat shock response for a conserved hypothetical protein that has GO terms associated with motor activity (PF3D7_0521900; PB-17), as well as the K13 propeller protein (PF3D7_1343700; PB-58), which has not previously been implicated in the heat shock response. K13 has been shown to bind protein substrates to facilitate ubiquitination and degradation; mutations in K13 associated with artemisinin resistance specifically disrupt K13’s binding to phosphatidyleinositol-3-kinase (PfPI3K), which leads to decreased proteasomal degradation of PfPI3K (16). Dysregulation of PfPI3K protein levels may also have consequences for redox (to which HSPs are also responsive) (17) and secretion of proteins important for host cell remodeling (18). Disruption of either of these pathways could have consequences for the response to febrile temperatures. Further study is required to investigate the link between K13 and the heat shock response.

Interestingly, one mutant had an enhanced response to heat shock in comparison to the wild type, though it displays growth attenuation under normal conditions (PB-43; Fig. 4). PB-43 has a disruption in the 3′ UTR of the apicoplast-targeted SufE-encoding gene (PF3D7_0206100). SufE is a protein known to be involved in the synthesis of iron-sulfur clusters, which act as important cofactors for several proteins, including those involved in stress responses (19, 20). It is tempting to speculate that this insertion somehow increases the availability of iron-sulfur clusters (perhaps through upregulation or increased transcript stability of SufE as a consequence of insertion in the 3′ UTR), which could increase the efficiency of HSPs aiding unfolded proteins resulting from heat stress. To our knowledge, the SufE pathway has not been implicated in the fever response before. Further studies are needed to confirm the transcript and protein expression consequences of this insertion. In addition, the potential importance of mitogen-activated protein kinase (MAPK) signaling pathways was revealed in this small phenotypic screen. Although the MKP-1 mutant PB-2 also has a growth defect, the link to the parasite’s survival response to febrile temperatures was confirmed in the phenotype rescue by genetic complementation.

The successful adaptation of experimental analytical methods for phenotypic screens for virulence factors opens the way for forward-genetic screens of *piggyBac* mutants. The approaches used in this proof-of-concept study relied on the assessment of the phenotypes of individual mutant clones. However, these assays were designed to be scalable for larger screens of *piggyBac* mutant parasite libraries. With the recent development of QIseq, the transition to phenotypic screens of mixed mutant libraries is now feasible. The advent of advanced forward-genetic screening tools for *P. falciparum* is an important step toward deciphering its genome to improve the development of new antimalarial therapies.

## MATERIALS AND METHODS

### Parasites and culture conditions.

*P. falciparum* NF54 *piggyBac* mutants were previously made in the course of a whole-genome random mutagenesis project (11). Briefly, mature blood-stage parasites were purified on a MACS magnetic column (Miltenyi Biotec) and 1 million purified parasites were added to erythrocytes loaded with 100 µg of a plasmid containing the *piggyBac* transposon with the human dihydrofolate reductase-encoding gene flanked with *P. falciparum* UTRs and 50 µg of the transposase plasmid to start a 5-ml parasite culture. Individual mutant clones were obtained by limiting dilution of parasites after drug selection. All insertion sites were identified by TAIL PCR as previously described (11), as well as by QISeq (Table 1) using the *P. falciparum* 3D7 genome as a reference (version 9.0, available from http://www.plasmodb.org). Insertions falling within 500 bp upstream of the translation start site were classified as 5′ UTR insertions. Insertions falling within 500 bp downstream of the stop codon were classified as 3′ UTR insertions and verified against the 3′ UTR data reported in reference 11. Several mutants were additionally verified via WGS (Table 1); these genome sequences have been deposited at http://www.plasmodb.org. All parasite cultures were maintained according to standard methods with gassing (5% O_2_ and 5% CO_2_, nitrogen balanced) and 5% hematocrit (O^+^ blood; Interstate Blood Bank) in RPMI 1640 medium (Invitrogen) supplemented with 0.5% Albumax II (Invitrogen), 0.25% sodium bicarbonate, and 0.01 mg/ml gentamicin. Cultures not undergoing heat shock were maintained at 37°C.

The complemented *piggyBac* mutant was made previously (14). The complemented *piggyBac* mutant was maintained in medium with 2 µg/ml blasticidin D to maintain the episome.

### Heat shock assays.

Figure 1 presents an overview of the heat shock assay protocol. After moderate parasitemia (1 to 3%) was reached, the cultures continued to grow in 5-ml flasks with complete medium at 5% hematocrit. Cultures were synchronized three times at 5% parasitemia with 5% sorbitol to ensure homogeneity. Parasites were first synchronized during the 10- to 12-h ring stage, again 48 h (one cycle) later, and for the third time 4 to 6 h later. The parasitemia was equalized to 3% for all cultures via microscopy, and they were diluted when necessary. For each biological replicate, three 96-well plates were prepared: (i) a *T*_0_ control plate (time zero, the start of the experiment, with parasites at the 16- to 18-h ring stage), (ii) a *T*_30_ control plate kept at 37°C for 30 h after the start, and (iii) a *T*_30_ plate heat shocked at 41°C for 8 h and then moved to 37°C for the remaining 22 h. Two hundred microliters each of three to five *piggyBac* mutant cultures, one NF54 wild-type culture, one uninfected RBC control, and one phosphate-buffered saline (PBS)-only control was added in triplicate wells to each of the three plates.

All plate samples were fixed in 200 µl of 0.05% glutaraldehyde at the conclusion of their respective time points. Plates were stored at 4°C for at least 1 h until preparation for flow cytometry.

### Flow cytometry.

Flow cytometry protocols for assessing *P. falciparum* parasitemia were conducted as previously described (11). Briefly, fixed samples in each 96-well plate were removed from 4°C storage. The supernatant was removed after centrifugation (450 × *g* for 3 min), and pellets were permeabilized in 0.3% Triton X-100 for 10 min and then washed in PBS. Next, samples were incubated for 1 h at 37°C in 0.5 mg/ml RNase A, followed by 1 h of staining with 0.1 mg/ml ethidium bromide at 37°C. The plates were then centrifuged, and the pellets were resuspended in PBS for Accuri C6 Flow Cytometer analysis directly from the 96-well plate. This system uses a 485-nm laser that measures forward light scatter to count cells and emission signals in the FL1 and FL2 channels (2). Infected cells were differentiated from uninfected cells by gating, where intense signals seen in populations in the FL2 channel for ethidium bromide or DNA stains indicated infected cells (Fig. 1). In each well, 50,000 cells were counted and the data were analyzed with CFlow Sampler software.

### Heat shock data analysis.

As *T*_0_ represents 16- to 18-h rings, the 30-h assay is largely confined to a single generation and therefore only measures growth (not new invasion). Only schizont-stage parasitemia was used to determine fold change. Changes in schizont parasitemia between 37°C and 41°C were acquired for mutant and wild-type cultures to measure variations in growth in response to heat shock. As several mutant parasite lines exhibit growth inhibition compared to the wild type under normal culture conditions, each mutant’s growth at 41°C was first standardized against its own growth at 37°C before comparison to the wild type by dividing schizont parasitemia for each technical replicate well at 41°C by average schizont parasitemia at 37°C and then averaging across biological replicates. The fold change for each parasite line was then divided by the wild-type value and plotted (Fig. 1; Fig. 2A). Data were assessed by one-way ANOVA of normalized log values and Dunnett’s multiple-comparison test on Prism software (GraphPad). Samples with a *P* value of 0.05 were considered significant.
